# Endocervical Carcinogenesis and HPV Vaccination: An Occasional Circumstance or a Gap in the Chain?

**DOI:** 10.1155/2017/4976741

**Published:** 2017-01-01

**Authors:** Georgios-Marios Makris, Petros Karakitsos, Eugenia Kotsifa, Niki Margari, Nikiforita Poulakaki, Theodoros N. Sergentanis, Marco-Johannes Battista, Charalampos Chrelias, Nicolaos Papantoniou

**Affiliations:** ^1^Gynecological Oncology Unit, Third Department of Obstetrics and Gynecology, General University Hospital “Attikon”, University of Athens, Chaidari, 12462 Athens, Greece; ^2^Department of Cytopathology, “ATTIKON” University Hospital, University of Athens Medical School, 1 Rimini, Chaidari, 12462 Athens, Greece; ^3^Department of Hygiene, Epidemiology and Medical Statistics, University of Athens Medical School, M. Asias 75, 11527 Athens, Greece; ^4^Department of Gynecology and Obstetrics, University Hospital of Mainz, University of Mainz Medical School, 55131 Mainz, Germany

## Abstract

As a result of the Human Papillomavirus (HPV) vaccination program, the prevalence of precancerous dysplasia and invasive cervical cancer has substantially decreased. In this brief report, we present a case of a young patient who was diagnosed with in situ adenocarcinoma of the cervix. This 30-year-old female had completed the HPV vaccination after she became sexually active and has been undergoing annual gynecological assessments, including clinical examination and Pap test, all of which had been negative. This year, her Pap test revealed a low grade squamous intraepithelial lesion (LGSIL) and additionally a colposcopy was performed. Given the extent of the lesion and since the colposcopy was inadequate, the patient underwent a type 3 large loop excision of the transformation zone and a curettage of the endocervix under local anesthesia. The pathological diagnosis from cervical biopsy revealed an in situ adenocarcinoma of the endocervix with negative limits. The HPV subtypes 16 and 83 were detected with PCR. After proper consultation she decided to preserve her fertility and to undergo a regular follow-up, postponing hysterectomy after the completion of her family planning. In conclusion, this case report highlights the need for diagnostic surveillance regarding HPV-related cervical cancer even after vaccination.

## 1. Introduction

Human Papillomavirus (HPV) is a DNA virus, member of the papillomavirus family, which causes genital warts, precancerous dysplasia, and cervical, anal, and other anogenital and oropharyngeal cancers. HPV infection is the most common sexually transmitted infection with prevalence of up to 80% among sexually active individuals of both sexes [1]. Genital HPV types fall into two categories: low-risk viruses that mainly cause skin lesions (condylomata acuminata) and high-risk viruses that are considered oncogenic. From the first group, the subtypes 6 and 11 account for 90% of all genital warts and from the second, HPV 16 and 18 are responsible for about 70% of cervical cancers [1, 2]. In 2006, a quadrivalent vaccine was licensed containing virus-like particles (VLPs) for HPV types 6, 11, 16, and 18. Later, in 2007, a bivalent vaccine became available offering protection against the subtypes 16 and 18 [3]. Since then, a variety of studies have proved that HPV vaccination is an effective strategy for reducing the impact of HPV-related diseases [4]. This is a case report of a patient with completed HPV vaccination and nevertheless diagnosed with an in situ adenocarcinoma caused by an HPV 16 infection.

## 2. Case Report

This is the case of a 30-year-old sexually active nulligravida female presenting with an abnormal Pap test. The patient had a free gynecological and family medical history, she has been smoking since the age of 15 (totally 10 py), and she had completed the HPV vaccination (three doses of the HPV vaccine) 6 years ago, after she became sexually active. There was no known history of STDs reported from her sexual partners (six in total) and she had the same sexual partner for the last 3 years.

The patient had been undergoing gynecological assessment by her treating specialist, including clinical examination and Pap test in a private practice, annually, since the age of 20, and all of them had been negative; however, at the age of 29, the Pap test revealed for the first time a low grade squamous intraepithelial lesion (LGSIL). One year later, the patient was referred to the Cervical Pathology Department, Third Department of Obstetrics and Gynecology, General University Hospital “Attikon”, University of Athens; after the clinical and gynecological examination, liquid phase cytological examination and a colposcopy were performed. The cytological assessment revealed a high-grade squamous intraepithelial lesion (HGSIL) and the PCR HPV test (CLART® Human Papillomavirus 2, GENOMICA) was positive for types 16 and 83. The dual staining immunocytochemical evaluation, using the CINtec® cytology double staining (p16/Ki67) kit, revealed p16 and ki-67 positivity.

Because of the extent and the immunocytochemical features of the lesion, and since the colposcopy was inadequate (the transformation zone was type 3 and the squamocolumnar junction was not entirely visible), the patient underwent a type 3 large loop excision of the transformation zone (LLETZ) and a curettage of the endocervix under local anesthesia. The pathological diagnosis from cervical biopsy revealed an in situ adenocarcinoma of the endocervix with negative limits. HPV typing in the biopsy sample confirmed the positivity for HPV 16 and 83 was detected. In addition, there was positive immunostaining of the protein p16 and Ki-67 expression was 30%.

After the counseling, the patient decided to preserve her fertility since she wishes to procreate. She was informed about the possible risks and decided to perform a hysterectomy by the completion of her family planning. One year after LLETZ, she remains without evidence of local recurrence or invasive cancer. Written informed consent was obtained by the patient for the publication of this case report.

## 3. Discussion

We present a case of cervical adenocarcinoma in situ (AIS) in a 30-year-old female with a positive HPV 16 typing test though fully vaccinated against HPV after the onset of her sexual life. This is a rare incident that is raising many questions about the efficacy and the safety of HPV vaccines. What remains unknown about this case is the time of the infection since the patient's annual Pap smears had been negative. One possible explanation may therefore be the efficacy of the vaccine. Since the two vaccines became available and were included in the National Vaccination Program of many countries including Greece, a variety of studies have been published concerning the impact of the vaccines on the prevalence of HPV 16 and 18 related precancerous lesions, in situ carcinomas, and invasive cancers.

Villa et al. [5] in the 5-year follow-up study performed in 2006 indicated that among quadrivalent vaccine recipients there was one case of HPV 16 DNA detection reported at the last visit before loss to follow-up and one case of verifiable persistent infection caused by HPV 18 infection. For this subject, the aforementioned subtype was detected only at months 12 and 18.

In 2007 the members of the FUTURE II study group [6] published a study based on a combined analysis of four randomized clinical trials. In this study, one case of HPV 16-related CIN3 was reported among vaccine recipients. Yet in this case, the patient was also tested positive for HPV 52. This specific subtype was detected at baseline and in five histology specimens, whereas HPV 16 DNA was detected in one histology specimen only during the diagnosis.

Wong et al. [7] presented some interesting data in 2010. Four cases of in situ and microinvasive cervical cancer were reported, identified in the Vaccine Adverse Event Reporting System (VAERS) database from January 1, 2006, through April 9, 2009. As far as the HPV subtype is concerned, three cases were identified as having high-risk HPV types, including one case with HPV 16. Nevertheless, the writers underlined that they did not have information on prevaccination HPV status of the patients in question (other than a single case, who was HPV positive prior to vaccination) and therefore exposure before vaccination could not be excluded.

In 2012, Szarewski et al. [8] identified five HPV-vaccinated patients with CIN III associated with HPV types 16 (three cases) and 18 (two cases) at the study endpoint. What is interesting about this study is that the women participating were included regardless of their HPV DNA, serological, and cytological status at baseline. For that reason it was possible to evaluate the vaccine efficiency (VE) in both seropositive and seronegative females. As it appears, in women with serological evidence of previous-to-vaccination HPV-16/18 infection, the VE was generally lower than in women who, at baseline, were either HPV DNA negative and seronegative or DNA negative regardless of serological status.

In another study by Gertig et al. [9] there are 47 cases of CIN3/AIS reported at the fully vaccinated cohort from which only one refers to adenocarcinoma in situ. In 2015, Apter et al. [10] reported in the PATRICIA trial (Papilloma TRIal against Cancer In young Adults) three cases of CIN1 in the vaccinated cohort and one case of CIN2 in a young woman. However, the patient in question acquired the HPV-16 responsible for development of the lesion before she completed the full three-dose series.

The aforementioned studies may indicate that the VE may not always be 100%. Therefore, it might be helpful to monitor the immunological response of the patient after the vaccination by controlling the antibodies against the HPV viruses, although the clinical relevance of this test has not been fully established. In conclusion, vaccinated females should not consider themselves totally secure. It is highly recommended that they continue their annual gynecological examination, since the Pap smear and/or HPV typing have been proved an excellent method of secondary prevention.

## References

[1] Weaver B. A. (2006). Epidemiology and natural history of genital human papillomavirus infection. *The Journal of the American Osteopathic Association*.

[2] Crosbie E. J., Einstein M. H., Franceschi S., Kitchener H. C. (2013). Human papillomavirus and cervical cancer. *The Lancet*.

[3] WHO (2009). Human papillomavirus vaccines. WHO position paper. *Weekly Epidemiological Record*.

[4] Brotherton J. M. L., Ogilvie G. S. (2015). Current status of human papillomavirus vaccination. *Current Opinion in Oncology*.

[5] Villa L. L., Costa R. L. R., Petta C. A. (2006). High sustained efficacy of a prophylactic quadrivalent human papillomavirus types 6/11/16/18 L1 virus-like particle vaccine through 5 years of follow-up. *British Journal of Cancer*.

[6] Ault K. A. (2007). Effect of prophylactic human papillomavirus L1 virus-like-particle vaccine on risk of cervical intraepithelial neoplasia grade 2, grade 3, and adenocarcinoma in situ: a combined analysis of four randomised clinical trials. *Lancet*.

[7] Wong C., Krashin J., Rue-Cover A. (2010). Invasive and in situ cervical cancer reported to the vaccine adverse event reporting system (VAERS). *Journal of Women's Health*.

[8] Szarewski A., Poppe W. A. J., Skinner S. R. (2012). Efficacy of the human papillomavirus (HPV)-16/18 AS04-adjuvanted vaccine in women aged 15–25 years with and without serological evidence of previous exposure to HPV-16/18. *International Journal of Cancer*.

[9] Gertig D. M., Brotherton J. M. L., Budd A. C., Drennan K., Chappell G., Saville A. M. (2013). Impact of a population-based HPV vaccination program on cervical abnormalities: a data linkage study. *BMC Medicine*.

[10] Apter D., Wheeler C. M., Paavonen J. (2015). Efficacy of human papillomavirus 16 and 18 (HPV-16/18) AS04-adjuvanted vaccine against cervical infection and precancer in young women: final event-driven analysis of the randomized, double-blind PATRICIA trial. *Clinical and Vaccine Immunology*.

